# Influencing Factors of Mobile Health Apps in Kidney Transplant Care: Systematic Review Using the Consolidated Framework for Implementation Research

**DOI:** 10.2196/84139

**Published:** 2026-03-24

**Authors:** Yingtian Jia, Shaobo Guo, Xinran Yang, Xiaohong Lin, Jiaxin Fang, Lei Dong, Xiangru Li, Haiya Sun, Wanhui Yu, Hongxia Liu

**Affiliations:** 1 School of Nursing, Beijing University of Chinese Medicine, No. 11 Beisanhuan East Road, Chaoyang District, Beijing, 100029, China, 86 15810116205; 2 School of Traditional Chinese Medicine, Beijing University of Chinese Medicine, Beijing, China; 3 School of Nursing, Jining Medical College, Jining, China

**Keywords:** mobile health, mHealth, kidney transplant, nursing care, influencing factors, systematic review, Consolidated Framework for Implementation Research, CFIR

## Abstract

**Background:**

Kidney transplant recipients require lifelong self-management and follow-up care to maintain allograft function. Mobile health (mHealth) effectively improves self-management behaviors and clinical indicators, consequently enhancing nursing care quality. However, these apps commonly face challenges, including low adoption rates and high discontinuation. Although researchers have explored associated facilitators and barriers from various perspectives, a systematic review of these influencing factors is lacking.

**Objective:**

The objective of this study was to systematically review the influencing factors of mHealth apps in kidney transplant care and to provide evidence for developing targeted interventions.

**Methods:**

The systematic review followed the PRISMA (Preferred Reporting Items for Systematic Reviews and Meta-Analyses) guidelines, and the protocol was registered in PROSPERO (International Prospective Register of Systematic Reviews, CRD420251091361). PubMed, Web of Science, Embase, MEDLINE, and Chinese databases, including China National Knowledge Infrastructure, Wanfang Data, China Science and Technology Journal Database, and SinoMed, were searched from inception to March 2025. The Mixed Methods Appraisal Tool was used for quality assessment given its suitability for appraising diverse study designs. Influencing factors were identified and coded according to the Consolidated Framework for Implementation Research due to its utility in systematically identifying multilevel implementation factors.

**Results:**

A total of 19 studies (all English-language publications) were included, comprising 9 qualitative studies, 5 mixed methods studies, and 5 quantitative studies, involving 1265 kidney transplant recipients and 34 health care providers. A total of 16 facilitators and 14 barriers were identified and categorized into 5 domains: intervention characteristics, outer setting, inner setting, characteristics of individuals, and process.

**Conclusions:**

The use of mHealth apps in kidney transplant care is influenced by multidimensional factors, with intervention characteristics constituting the most prominent domain, while the outer setting and process domains are relatively underrepresented. Future research should investigate these influencing factors and implement multidimensional strategies to optimize mHealth apps in kidney transplant care.

## Introduction

Kidney transplantation is currently the most effective treatment for end-stage renal disease, effectively alleviating clinical symptoms and prolonging patient survival [1,2]. Research indicates that the 1-year survival rate for kidney transplant recipients has reached 97.8%, with the 5-year survival rate at 88.1% and the 10-year survival rate at approximately 60% [2]. However, due to the risks of graft rejection and long-term immunosuppressant use, kidney transplant recipients require lifelong self-management and follow-up care to maintain allograft function [3].

The World Health Organization defines mobile health (mHealth) as "medical and public health practice supported by mobile devices, including mobile phones, patient monitoring devices, personal digital assistants, and other wireless technologies” [4]. Currently, mHealth has been applied across multiple domains of kidney transplant care, including medication adherence reminders [5], weight management [6], and postoperative self-monitoring [7]. Studies demonstrate that mHealth interventions can effectively enhance self-management behaviors, improve treatment compliance, optimize clinical outcomes, and elevate nursing care quality [8-10]. Additionally, mHealth apps can reduce transportation costs and time burdens for recipients, mitigate disease exposure risks, and decrease nosocomial infection rates [11,12]. Nevertheless, challenges persist, including low software adoption rates, high discontinuation rates, and short user retention periods [13]. Existing research has explored facilitators and barriers from the perspectives of device functionality, user experience, and management protocols [11,14,15], yet it lacks a comprehensive synthesis of these influencing factors.

The Consolidated Framework for Implementation Research (CFIR) was developed by Damschroder et al [16]. As a widely recognized and extensively cited framework in implementation science, it is applicable to all implementation stages, including preimplementation, active implementation, and sustainability [17]. This theoretical framework encompasses 5 domains (intervention characteristics, outer setting, inner setting, characteristics of individuals, and process) and 39 underlying constructs. It enables systematic identification of multidimensional and multilevel influences during intervention implementation processes. Guided by the CFIR framework, this study aims to systematically evaluate the influencing factors of mHealth apps in kidney transplant care, identify key facilitators and barriers, and provide evidence-based recommendations for developing targeted intervention strategies.

## Methods

### Search Strategy

A comprehensive computerized search was conducted across 8 English and Chinese databases: PubMed, Web of Science, Embase, MEDLINE, China National Knowledge Infrastructure, Wanfang Data, China Science and Technology Journal Database, and SinoMed, covering literature from inception to March 2025. The search focused on identifying influencing factors of mHealth apps in kidney transplant care. Search terms were structured using a combination of Medical Subject Headings and keywords. English search terms: “kidney transplant* OR renal transplant* OR kidney graft* OR renal graft* OR kidney allograft* OR renal allograft* OR transplanted kidney OR kidney homotransplantation* OR renal homotransplantation*,” “tele?medicine OR telemedicine OR mobile health OR m?Health OR mHealth OR tele?health OR telehealth OR e?Health OR eHealth OR tele?rehabilitation OR telerehabilitation OR tele?monitoring OR telemonitoring OR video?conferencing OR videoconferencing OR online OR technolog* OR mobile information technolog* OR electronic OR digital OR Internet-based OR app* OR mobile app* OR software OR wearable electronic device* OR WeChat OR platform* OR smartphone*,” “motivator* OR motivation* OR enabler* OR promote OR drive OR encourage OR facilitator* OR barrier* OR obstacle* OR challenge* OR difficult* OR issue* OR influencing factor* OR impact factor* OR relative factor* OR experience* OR perception* OR opinion* OR feeling* OR need* OR attitude*.” The search strategy was adjusted according to different databases. Key adaptations included the use of database-specific subject headings (eg, Medical Subject Headings in PubMed, Emtree in Embase) and adjustments in field tags (eg, [Title/Abstract] in PubMed; ab, kw, ti in Embase). Taking the PubMed database as an example, the search query is provided in Multimedia Appendix 1. To ensure comprehensive reporting, the PRISMA checklist is provided in Checklist 1.

### Inclusion and Exclusion Criteria

Inclusion criteria:

1. Study subjects: kidney transplant recipients and stakeholders involved in mHealth apps (eg, health care providers, caregivers);
2. Study content: facilitators, barriers, or influencing factors related to the application of mHealth, with mHealth functions relevant to transplant care;
3. Study design: qualitative, quantitative, or mixed methods studies.

Exclusion criteria:

1. Conference abstracts, unpublished data, or inaccessible full texts;
2. Secondary literature (reviews, systematic reviews) or duplicate publications;
3. Non-Chinese or non-English publications.

### Literature Screening and Data Extraction

Two researchers trained in evidence-based nursing systematically and independently conducted the screening process using predefined protocols. Duplicates were removed using NoteExpress 3.8 software. Initial exclusion was performed through title and abstract screening, followed by full-text assessment. Discrepancies were resolved through consultation with a third researcher.

Data extraction was independently performed by 2 researchers. To ensure accuracy and completeness, all extracted data were cross-checked against the original full-text articles by a third researcher. Any disagreements or uncertainties were resolved through team discussion. The data extraction form was independently developed by the researchers, subsequently refined through research team discussions, and ultimately finalized for implementation. Extracted data included authors, publication year, country, study design, sample size, study population, sampling method, data collection methods or tools, names and functions of mHealth apps, and Mixed Methods Appraisal Tool (MMAT) rating.

### Quality Assessment

Given the heterogeneity of study designs, 2 researchers independently evaluated the methodological quality of included studies using the MMAT, 2018 edition [18]. Discrepancies were resolved through consensus discussions with a third researcher. The MMAT was developed by a research team at McGill University, Canada, in 2006 and updated in 2018 [18]. It is designed to appraise the quality of qualitative, quantitative, and mixed methods research studies. The tool comprises 2 screening questions and 15 appraisal criteria. After addressing the 2 screening questions, the appropriate study category is selected for appraisal. Qualitative and quantitative studies require the evaluation of 5 criteria each. Each item was rated as “Yes,” “No,” or “Unclear.” A rating of 5***** or 100% indicates fulfillment of all quality criteria, 4**** or 80% indicates fulfillment of 80% of the criteria, and so forth. For mixed methods studies, all 15 criteria (instead of 5) must be appraised. The overall quality rating adheres to the principle that the quality of the whole is determined by its weakest component.

### Data Synthesis

This study conducted data analysis anchored in the 5 domains of the CFIR [16] and adhered to the Joanna Briggs Institute guidelines [19] for mixed methods systematic reviews. The analytical process involved: (1) integrating findings from distinct research methodologies separately; (2) translating quantitatively synthesized results into qualitative narratives and merging them with qualitatively synthesized evidence; and (3) finally establishing integration categories, inducing thematic patterns, and deriving consolidated outcomes.

## Results

### Literature Search Results

**Figure 1.**
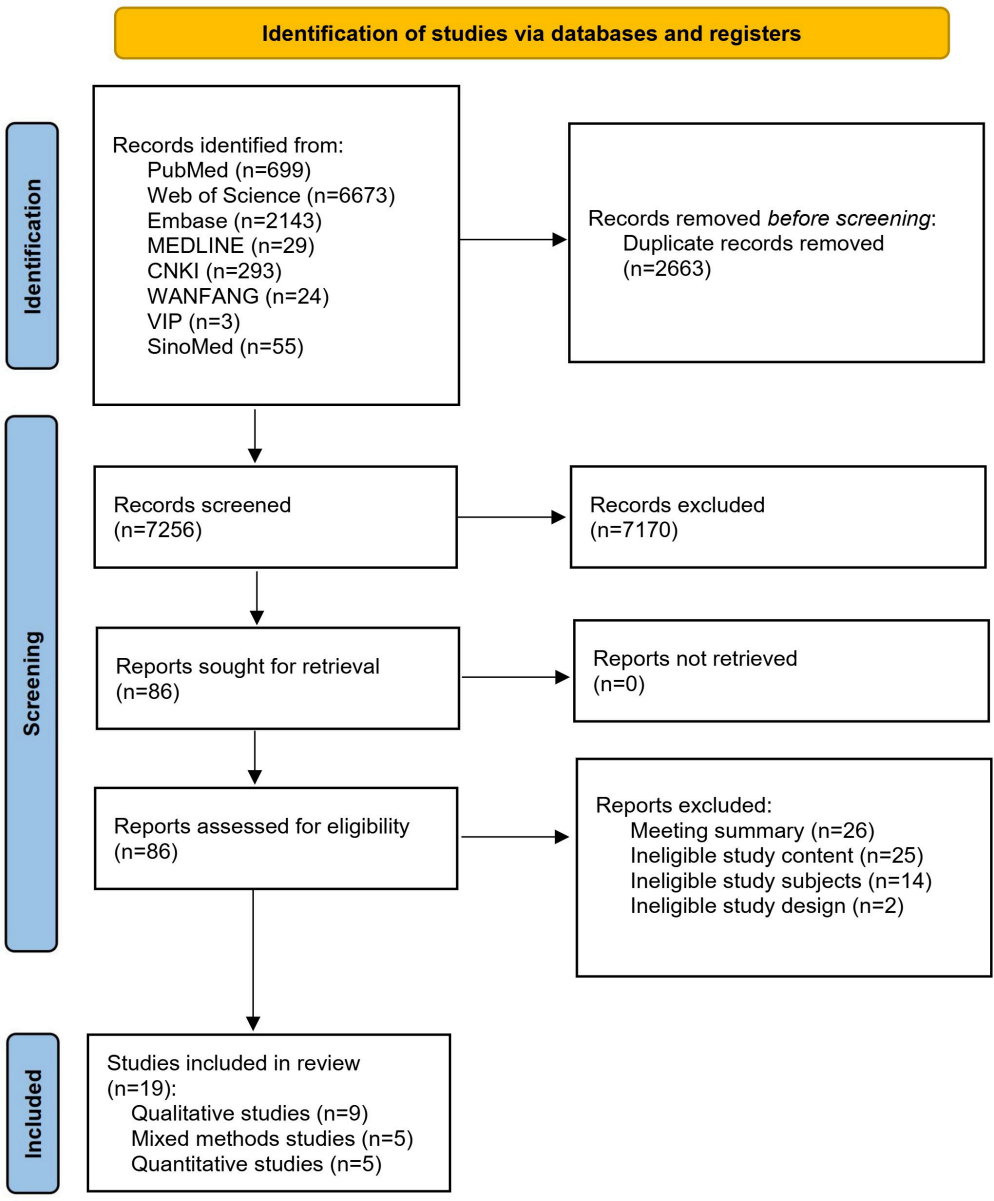
PRISMA (Preferred Reporting Items for Systematic Reviews and Meta-Analyses) flow diagram. CNKI: China National Knowledge Infrastructure; VIP: China Science and Technology Journal Database.

A total of 9919 relevant studies were retrieved from the databases. After removing duplicates, 7256 studies remained. Following preliminary screening, 86 studies were retained. Ultimately, 19 studies were included after full-text review. The literature selection process and the list of included studies are presented in Figure 1 and Table 1, respectively.

**Table 1.** Characteristics of the included studies.^a^

Author(s)	Year	Country	Study design	Sample size (n)	Study population	Sampling method	Data collection methods/tools	Names/functions of mHealth	MMAT rating
Esayed et al [11]	2025	United States	Qualitative	20	Adult kidney transplant recipients	Purposive sampling	Semistructured interviews	A health care delivery platform using a live video visits for post-transplant follow-up	5*****
Malo et al [20]	2024	Canada	Qualitative	11	Kidney transplant recipients participating	Purposive sampling	Semistructured interviews	KEeP ACTIVe Club for increasing physical activity levels and reducing loneliness in kidney transplant recipients	5*****
Tang et al [21]	2023	Australia	Mixed methods	91	Adult kidney transplant recipients	Multistage sampling	Questionnaires (Self-developed questionnaire, eHealth Literacy Scale); semistructured interviews	eHealth	1*
Tang et al [22]	2022	Australia	Qualitative	30	Adult kidney transplant recipients	Purposive sampling	Semistructured interviews	eHealth	5*****
Castle et al [23]	2022	United Kingdom	Mixed methods	n_1_=17 (quantitative); n_2_=13 (qualitative)	Adult kidney transplant recipients	Unreported (quantitative); Purposive sampling (qualitative)	Questionnaires (General Practice Physical Activity Questionnaire, Nutrition Self-Efficacy Scale, Physical Exercise Self-Efficacy Scale, EuroQol 5-Dimension-5 Level, Chalder Fatigue Scale, 6-min walk test); body composition and parameter measurements; semistructured interviews	ExeRTion for prevention of post–kidney transplantation weight gain	2**
Huuskes et al [24]	2021	Australia	Qualitative	34	Adult kidney transplant recipients	Unreported	Focus group interviews	Telehealth	5*****
Castle et al [15]	2021	United Kingdom	Qualitative	17	Kidney transplant recipients (n=11), transplant-related health care providers (n=6)	Purposive sampling	Think-aloud method; semistructured interviews	ExeRTion for prevention of post–kidney transplantation weight gain	5*****
Krause et al [25]	2021	Germany	Mixed methods	55	Adult kidney transplant recipients	Convenience sampling	Questionnaires (4-item Basel Assessment of Adherence to Immunosuppressive Medication Scale, Self-developed questionnaire); blood assays; semistructured interviews	SimpleMed+ for estimating medication adherence in kidney transplant patients	1*
O'Brien et al [26]	2020	United States	Quantitative	53	Older kidney transplant recipients (≥60 y)	Convenience sampling	Questionnaires (Self-developed questionnaire)	Fitbit Charge 2 for collecting physical activity data in real time	4****
Nielsen et al [27]	2020	Denmark	Qualitative	36	Adult kidney transplant recipients (n=16), clinicians (n=16), nurses (n=4)	Purposive sampling	Semistructured interviews; focus group interviews	Telehealth solution consisted of a mobile app and a workflow for posttransplant follow-up	5*****
O'Brien et al [28]	2020	United States	Qualitative	20	Adult kidney transplant recipients	Purposive sampling	Semistructured interviews	mHealth apps	5*****
Côté et al [29]	2019	Canada	Qualitative	10	Adult kidney transplant recipients	Purposive sampling	Semistructured interviews	Web-based tailored virtual nursing intervention for promoting medication adherence and supporting self-management among kidney transplant recipients	5*****
Wedd et al [14]	2019	United States	Quantitative	710	Adult kidney transplant recipients (n=455) and liver transplant recipients (n=255)	Unreported	Medical records and database extraction	Web-based patient portal systems	3***
O'Brien et al [30]	2018	United States	Quantitative	165	Adult kidney transplant recipients	Convenience sampling	Questionnaires (Self-developed questionnaire)	Mobile apps for self-management of care among kidney transplant recipients	4****
Wang et al [31]	2017	Netherlands	Quantitative	46	Adult kidney transplant recipients	Unreported	Questionnaires (Self-developed questionnaire)	Self-management support systems (SMSS) for increasing the level of self- management among kidney transplant recipients	4****
Van Lint et al [32]	2015	Netherlands	Mixed methods	30	Adult kidney transplant recipients	Unreported	Questionnaires (Self-developed questionnaire, Worry Scale, Transplant Effects Questionnaire, Self-efficacy Scale, Health Care Climate Questionnaire); semistructured interviews	StatSensor Xpress for creatinine self-measurement; Microlife WatchBP Home for blood pressure self-measurement; disease-management system (DMS) for registering measurement results	5*****
McGillicuddy et al [33]	2013	United States	Quantitative	99	Adult kidney transplant recipients	Convenience sampling	Questionnaires (Self-developed questionnaire, Perceived Stress Scale, 7-item Morisky Medication Adherence Scale)	A mHealth remote monitoring system for monitoring medication adherence and physiological parameters	5*****
Schäfer-Keller et al [34]	2009	Switzerland	Qualitative	22	Adult kidney transplant recipients (n=14), clinicians (n=8)	Purposive sampling	Structured interviews	OTIS for increasing the level of self-management among kidney transplant recipients	5*****
Russell et al [35]	2009	United States	Mixed methods	85	Older kidney transplant recipients (>55 y)	Convenience sampling	Questionnaires (Self-developed questionnaire); semistructured interviews	MEMS for improving medication adherence among kidney transplant recipients	1*

a The Mixed Methods Appraisal Tool (MMAT) uses the following rating system: 5***** indicates full compliance with 100% of the quality assessment criteria, 4**** corresponds to meeting 80% of the criteria, 3*** signifies adherence to 60%, 2** represents 40%, and 1* denotes meeting 20% of the standards.

### Characteristics of the Included Studies

All included studies were published in English, with publication years spanning from 2009 to 2025. These studies originated from 8 countries, with the United States contributing the highest number of publications [11,14,26,28,30,33,35]. The research designs comprised 9 qualitative studies, 5 mixed methods studies, and 5 quantitative studies. The study populations included 1265 kidney transplant recipients and 34 health care professionals. The basic characteristics of the included studies are summarized in Table 1.

### Quality Assessment of the Included Studies

A total of 9 qualitative studies exhibited high overall quality, each scoring 5*****. Among the 5 quantitative studies, 1 [14] did not clearly describe whether the sampling method was appropriate for addressing the research questions, another [14] failed to specify the representativeness of the sample relative to the target population, 1 [30] lacked clarity on the risk of nonresponse bias, 1 [26] did not explicitly address confounding factors in the study design or analysis, and 1 [31] did not confirm whether interventions were implemented as intended during the research period. The 5 mixed methods studies demonstrated lower overall quality: 4 studies [21,23,25,35] did not justify the rationale for adopting a mixed methods design to address the research questions, 4 [21,23,25,35] inadequately explained the integration of qualitative and quantitative results, 4 [21,23,25,35] insufficiently addressed discrepancies or heterogeneity between quantitative and qualitative findings, and 3 [21,25,35] failed to cohesively integrate distinct components of their research. The MMAT scores for all included studies are presented in Table 1.

### Factors Influencing the Application of mHealth in Kidney Transplant Care

This study identified 16 facilitators and 14 barriers influencing the application of mHealth in kidney transplant care. Based on the CFIR, both facilitators and barriers were categorized into 5 domains, as detailed in Table 2.

**Table 2.** Factors influencing the application of mHealth in kidney transplant care.

Category	Facilitators	Barriers
Intervention characteristics	Evidence strength and quality: Authoritative content [21,22] Relative advantage: Convenience and flexibility (time-, effort-, and cost-saving) [11,21,22,24,27,29,35] Reduced infection risk [11,22,24,31] Improved comfort [11] Design quality and packaging: User-friendly design (easy to understand, operate, and humane) [22,28,29,35] Personalized design [15,22,28]	Evidence strength and quality: Lack of authoritative content [34] Complexity: Communication/interaction limitations [11,20,22,24,27] Privacy/security risks [22,23] Negative online content [22] Design quality and packaging: Poor design (complex operation, unclear content, inappropriate sizing) [15,21,25,26,28,30,34,35]
Outer setting	Patient needs and resources: No additional costs [33]	Patient needs and resources: External environmental interference [23,24] Extra costs [30]
Inner setting	Networks and communications: Professional support [23,32] Peer support [15,20,21]	Structural characteristics: Unstable network/equipment [24] Networks and communications: Lack of multidisciplinary team support [24] Compatibility: Time conflicts [20] Conflicts with user habits [25,35]
Characteristics of individuals	Knowledge and beliefs about the intervention: mHealth literacy [14,21] Self-efficacy: Self-efficacy [23,32] Individual identification with organization: Perceived benefits [20,21,29,31,34] Other personal attributes: Intrinsic motivation (curiosity, altruism, responsibility) [20,23,26,29] Demographics (race) [33]	Knowledge and beliefs about the intervention: Lack of mHealth literacy [21,22,24,30] Other personal attributes: Poor physical condition [24,30]
Process	Engaging: Training on device functions [15,30] Executing: SMS/email reminders [15]	Engaging: Lack of training on device functions [27]

### Facilitators

#### Intervention Characteristics

Intervention characteristics are critical predictors of the successful application of interventions. The authoritative content of mHealth platforms can effectively alleviate user concerns and enhance their confidence in using these tools (“These sorts of webpages would need to carry some sort of certification so that we, the individuals, know we are getting professional advice as opposed to some blogger.” [21]). Compared to traditional face-to-face care models, mHealth offers advantages such as convenience and flexibility ("Now [with telehealth] it’s very convenient for me because I can even take calls while I’m working in the office or whether I’m home.” [24]; “I think video visits might actually help with compliance, especially for socioeconomically disadvantaged or older patients that don’t drive.” [11]), reduced infection risks (“I prefer [eHealth], I’m so scared of COVID. I don’t want to get sick, so I’m happy to do the online, video calls and things like that.” [22]), and enhanced comfort ("I was comfortable sharing personal information since, like I said, I’m in my own house. So, it’s easier to actually sometimes talk to the doctor when you’re at home.” [11]). These advantages serve as key motivators for users to persist with mHealth. Additionally, some users emphasized that designs featuring clear language (“Level of language, get the language to a layperson’s point of view. Doctors would understand what it was and so would the person who is reading it… [or else] they would go away from it.” [22]), ease of use (“You know, it’s user-friendly, going to it isn’t complicated. […] It’s pretty easy to figure out. […] You quickly get the hang of it.” [29]), human-centered care ("The nurse came across as very professional […] as someone who cares about your health […] Me, I found her to be warm. Even though it was a video, it’s. it’s not. it can seem a little cold, but it’s someone who’s talking to us, deep down.” [29]), and personalization (“If something was going wrong, yes, rather than just getting an automatic message that was just saying ‘keep going for your goals!’ and you’re like ‘well I haven‘t been on 3 weeks’. I would prefer something more personable.” [15]) would make mobile devices more appealing.

#### Outer Setting

Users’ needs and resource accessibility are critical external factors determining the adoption of mHealth. If mHealth solutions require no additional financial burden on users, their willingness to adopt is significantly enhanced (79% of users endorsed this view [15]).

#### Inner Setting

Multidisciplinary collaboration and communication are central to the successful application of mHealth. Regular communication and professional guidance from health care providers motivate patients to adhere to care plans (“I think, um, it will be helpful [for my adherence] because I spoke to my physio quite a bit. She used to call me and, um, she would—I’d tell her sometimes—and she’d be like, you know what, you know keep busy, do this and do that and stuff like that. She would give me advice.” [23]). Some patients highlighted that sharing experiences and emotional support with peers fosters a sense of connection, alleviates loneliness, and strengthens their resolve to persist (“When I had a question, I could send a text and they [peers] responded right away, right away. So it was really good, you know.” [20]; “Being together, that always motivates more than when we’re alone.” [20]).

#### Characteristics of Individuals

Individual characteristics are pivotal factors influencing the adoption of mHealth. An individual’s mHealth literacy directly impacts the effectiveness of mHealth use. Those with higher mHealth literacy exhibit stronger technical proficiency, information evaluation skills, and greater acceptance of mHealth (“Factors associated with mHealth use include higher eHEALS scores and higher education, with odds ratios [ORs] of 1.21, 95% CI 1.06‐1.38 and 7.78, 95% CI 2.19‐27.7, respectively.” [21]). Self-efficacy reinforces individuals’ confidence in using mHealth (“I was just [pause] following the program through. Um, but that was just my personal thing, just because I have—you know—I have the knowledge and the confidence to do my own thing.” [23]). Some patients noted that using mHealth offers benefits such as enhanced self-management (“Patient portal containing access to all of the above suggestions in one unified system, where my blood results, education, and app monitoring can all be tracked by myself at a click of a button. This will ultimately increase self-management among patients and allow for more detailed ‘healthier life’ conversations with health professionals.” [21]) and disease-related knowledge (“All important things are included [in this platform]: where I have to take precautions, what I need to do. Everything is well explained.” [34]). Intrinsic motivations such as curiosity (“It was out of curiosity. I’m naturally curious. So I really wanted to see how far it went...” [29]), altruism (“Well, I did it for you… it was to help you out, because you help me in a way. Your aim… the reason that you’re doing this, I imagine, is to help me.” [29]), and a sense of responsibility ("So, my point there is in terms of being accountable to something. Even though it’s not a human being, you are being accountable to a system…” [23]) drive patients to persist. Additionally, race influences attitudes toward mHealth (“Black individuals exhibited more positive attitudes than White individuals [mean 4.25, SD 0.88 vs mean 3.76, SD 1.07; *P*=.02].” [33]).

#### Process

Supportive strategies during the implementation process are critical factors for ensuring the sustainability of mHealth interventions. Participants emphasized that receiving training prior to using mHealth builds operational confidence and foundational knowledge, reducing adherence decline or misuse caused by technical unfamiliarity (“I think the first steps you need it face-to-face to start with… then you can do it on your own at home.” [15]). Additionally, reminder features in devices play a role in encouraging adherence (“If there’s a 12-week program, what would be useful would be a text reminder or an email reminder.” [15]).

### Barriers

#### Intervention Characteristics

Inherent limitations of mHealth may hinder its application. When mHealth platforms provide content lacking official certification or conflicting with clinical recommendations, patients may question its reliability (“My doctor advised taking tacrolimus between meals, but the platform instructed me to take it 1 h before or 1.5‐2 h after meals.” [34]). User-unfriendly designs, such as overly lengthy interfaces (“Instead of scrolling through all the different things because that’s [the excessively long pages] what drives me crazy with apps.” [28]) or bulky devices (“The mobile health device [MEMS] is too large to carry around or travel with.” [35]), foster resistance and reduce willingness to continue use. The virtual nature of mHealth limits nonverbal interaction (“I like face‐to‐face because my doctor tells me if he thinks that I’m putting on weight or I’m not doing the right thing.” [24]; “Telehealth can’t take your blood pressure… and they can’t take a look at your wound or check you or examine you or weigh you. Sitting down, I could have gained 25 kg, but no one would know.” [22]). Participants also expressed concerns about privacy breaches (“Hearing about hackers, and you don’t want your records and things like that when you subscribe to these sites and things. You’re putting yourself at risk and your whole medical history at risk. There are things in my medical history that I don’t want people to know.” [22]). Additionally, exposure to negative online content during use imposed psychological burdens (“Sometimes it got a bit depressing… it was like really a bit of a mental burden as well, just like constantly getting notifications about this message and that message and someone dying. Sometimes when you’re in a difficult headspace, it can be really hard to be confronted with that.” [22]).

#### Outer Setting

The inability of external resources to meet user needs can hinder the application of mHealth. The use of mHealth may be interfered with by external environments, thereby negatively impacting its effectiveness (“Because I think face‐to‐face, you’re in the room, you’re kind of in the moment, with telehealth, you can get distracted. You can get a bit confused” [24]; “If you’re doing telehealth, then you might miss something like the dog barking in the background or the kids screaming.” [24]). Additionally, participants noted that additional costs associated with mHealth reduce willingness to use such services, as these expenses impose financial burdens [30].

#### Inner Setting

Structural barriers within mHealth systems represent core constraints affecting their application. Participants reported that unstable network equipment compromises their user experience (“Like when the internet’s down, when it’s slow, which it is often at my place. Even my phones aren’t that good. The landline, even… [Whether I can use mHealth normally] just depends on if everything’s working.” [24]). Compared to traditional face-to-face clinical models, mHealth struggles to ensure patients receive simultaneous support from multidisciplinary teams (“When I do telehealth, you become sort of like missing out on all the other aspects of allied health.” [24]; “Nurses are a part of my training team as a nephrologist, and I feel that connect with the nurses is completely cut off [with telehealth].” [24]). Some mHealth services require advance appointments, yet users often fail to attend due to personal scheduling conflicts (“The schedule was a problem, it [mHealth] just didn’t work for me at all.” [20]). Additionally, some participants expressed difficulty adapting to new self-management models via mHealth due to entrenched habits (eg, medication or exercise routines) (“When the situation differs from my routine, it [mHealth] gets in the way and is hard to accommodate.” [35]).

#### Characteristics of Individuals

User characteristics directly influence the effectiveness of mHealth adoption. For users with limited mHealth literacy, such as older adults or those with lower education levels, technical challenges encountered during mHealth use often lead to abandonment of the technology (“Not being a tech-savvy person, I find trying to navigate and download apps very stressful.” [21]; “The problem is that when you get older, it’s harder for you to understand and pick up if you are not constantly on the computer. I don’t use it much.” [22]). Additionally, users’ physical conditions impact mHealth utilization, with patients reporting that visual impairments and limited hand dexterity hinder their engagement with mHealth platforms [30].

#### Process

Insufficient training on mHealth use results in ongoing challenges for users during later implementation ("I would say it is incredibly complex in the sense that when the doctor says it, it sounds logical and totally correct, but when you come home, then what? Because then if I make a slight deviation, is it the same, or is it a new situation?” [27]).

## Discussion

### Principal Findings

An important finding of this review is that, despite the comprehensive 5-domain analytical structure offered by CFIR, a notable imbalance exists in the focus of existing research. Influencing factors are predominantly concentrated within the domains of intervention characteristics, characteristics of individuals, and the inner setting. In contrast, factors related to the outer setting and process domains, such as policy support and stakeholder collaboration, remain significantly underrepresented.

This pattern may be explained by 2 primary reasons. First, the majority of included primary studies focused on technology acceptance and user experience. Their participants were predominantly kidney transplant recipients, who are generally not in a position to provide in-depth perspectives on macrolevel issues such as health care policies, regional resource distribution, or multistakeholder coordination. Second, evidence concerning the complex factors of the outer setting and process is more likely to reside in what is known as “grey literature,” including project evaluation reports, dissertations, or specific policy studies, which was not captured by our predefined academic database search.

### Optimizing mHealth Design and Performance to Enhance User Experience

The findings of this study indicate that the application of mHealth is closely tied to its inherent characteristics. The domain of intervention characteristics contains the highest number of influencing factors, which aligns with prior studies [36]. For successful integration into the workflow, the intervention needs to align with the prevailing value system of key stakeholders.

After enduring the hardships of dialysis, most kidney transplant recipients highly value their transplanted kidney and exhibit heightened caution in health management [37]. The authoritative nature of mHealth content helps build trust in the platform and strengthens users’ confidence. Studies [38,39] reveal that approximately 30% of kidney disease–related apps on the market lack clinically validated content, posing risks such as incorrect medication advice or deviations from the latest guidelines, which significantly undermine patient trust and hinder sustained use. Therefore, mHealth development should be grounded in evidence-based medicine and clinical guidelines, with regular updates. Prior to releasing content, experts in kidney transplantation should review materials to ensure scientific rigor. The design and performance of mHealth directly impact user experience. Kidney transplant recipients vary widely in age, educational background, and health literacy. With global population aging, the proportion of older recipients is rising [40,41]. Older users may struggle with complex medical terminology or operational workflows due to cognitive decline or limited technical adaptability. Thus, mHealth design should prioritize accessibility, using plain language and intuitive interfaces to lower barriers and improve acceptance. Kidney transplant recipients commonly face psychological challenges such as anxiety and depression [42]. Research [43] shows that integrating emotional support features into mHealth can alleviate patient anxiety and enhance long-term engagement. Additionally, lifelong self-management is essential for recipients. If apps lack personalized feedback, users may abandon them due to perceived low value. To address this, personalized features such as achievement badges to motivate users or tailored notifications to boost engagement could be incorporated.

mHealth is a double-edged sword. While offering advantages such as convenience, flexibility, and reduced infection risks, it also has limitations, including security risks, hindered clinician-patient interaction, and exposure to negative content, which diminish user willingness to adopt it. Studies [44,45] highlight widespread patient concerns about data security in mHealth, particularly for long-term conditions such as kidney transplantation, where sensitivity to privacy breaches is heightened [46]. Thus, health care providers should strengthen awareness of data protection and regularly debug and update software to prevent cyberattacks. Beyond safeguarding privacy, stakeholders could enhance user experience by improving content moderation and integrating wearable devices to mitigate mHealth’s shortcomings.

### Strengthening Multidimensional Support Systems to Improve Resource Accessibility

The findings of this study reveal that the adoption of mHealth is influenced by multiple internal and external factors. Additionally, outer setting factors are relatively few among all influencing factors. This aligns with a prior scoping review by Rangachari et al [47].

Peer, professional, and multidisciplinary team support significantly impact kidney transplant recipients’ engagement with mHealth, suggesting that health care providers should establish multidimensional support strategies. Peer support fosters emotional resonance and experience sharing among patients with similar backgrounds, reducing loneliness and enhancing willingness to use mHealth. Therefore, integrating peer communication modules into mHealth platforms is recommended; however, medical advice or medication sharing should be explicitly prohibited to ensure compliance. Guidance and feedback from health care professionals can alleviate patients’ technological apprehensions. Studies [48] indicate that lack of clinician support is a barrier to mHealth adoption, aligning with this study’s conclusions. Thus, remote follow-ups by professionals to guide patients, alongside accessible channels for reporting issues, are proposed to build trust and sustain engagement. The development and implementation of mHealth require collaboration and guidance from multidisciplinary teams. Without such support, unresolved technical or medical challenges during use may lead to unmet needs and reduced compliance [49]. Future initiatives should establish mHealth teams comprising physicians, specialist nurses, rehabilitation therapists, and IT engineers to optimize application effectiveness. Additionally, internal and external factors such as additional costs and unstable network infrastructure hinder mHealth accessibility. Expenses related to data consumption or smart device procurement may impose financial burdens on patients. Research [50] reports that 23% of patients in resource-limited settings abandon mHealth due to unaffordable device costs. To address this, governments and hospitals should establish subsidy mechanisms to reduce user expenses. Developing low-data or offline versions of mHealth platforms could simultaneously mitigate data consumption and connectivity issues, thereby improving resource accessibility.

### Stimulating Individuals’ Intrinsic Motivation, Enhancing Perceived Benefits, and eHealth Literacy

The findings of this study indicate that the application of mHealth is influenced by individual characteristics, such as intrinsic motivation and perceived benefits. Notably, factors at the individual level are relatively numerous among all identified influences, which is similar to the findings of the previous review by Neil-Sztramko et al [51].

Intrinsic motivation refers to an individual’s inherent tendency to seek novel challenges, develop competencies, and engage in exploratory learning driven by personal interest or enjoyment of the activity itself, which is more likely to sustain long-term behavioral adherence [52]. For users lacking intrinsic motivation, motivational interviewing techniques such as goal setting, reflective practices, verbal persuasion, and action planning can be used to initiate and reinforce their motivation, thereby promoting behavioral persistence [53]. This study also reveals that perceived benefits can enhance users’ sustained engagement with mHealth. Research demonstrates [54] that perceived benefits indirectly influence behavioral adoption through the mediating role of self-efficacy. Health care professionals should acknowledge and encourage users’ perceived benefits, reinforce positive experiences through multiple channels, and further enhance self-efficacy to facilitate long-term mHealth adherence. When users fail to perceive benefits, their behavioral persistence may be compromised [55]. In such cases, practitioners should promptly adjust intervention strategies through telephone follow-ups, video demonstrations, and other methods to strengthen perceived benefits and improve self-efficacy. Furthermore, this study identifies that insufficient eHealth literacy constitutes a barrier to mHealth utilization. Some users experience difficulties in independently operating smartphones or applications due to age, educational background, or limited digital skills, leading to resistance or abandonment of mHealth tools. Therefore, beyond simplifying the usability of mHealth systems during development, health care providers should strengthen preimplementation training on device functionalities. Personalized training programs tailored to users’ varying health literacy levels should be developed to compensate for eHealth literacy deficiencies.

### Limitations

#### Limitations of the Included Studies

The inclusion of studies with varied methodological quality, while ensuring comprehensive analysis, may have introduced potential bias. The lower overall ratings for some mixed methods studies primarily reflected limitations in their integrative components rather than in the qualitative or quantitative parts from which data were extracted. Future studies should be more methodologically rigorous and better designed to enhance the overall quality and reliability of the evidence base in this field. All included studies were published in English and conducted in developed countries, introducing both linguistic and geographic bias. Consequently, the findings primarily reflect contexts with abundant resources and well-developed digital infrastructure, which may limit the generalizability of the conclusions. It is imperative to conduct research focusing on developing countries and non–English-speaking regions. No subgroup analysis was conducted for key variables (eg, age, mHealth type). This was primarily because most studies did not stratify outcomes by age or treated mHealth as a broad concept without specifying functional types, hindering the extraction of subgroup-specific factors. Publication bias was not formally assessed. Due to the nature of this review, which included diverse study designs and focused on synthesizing influencing factors rather than quantifying a single effect size, standard graphical tests for publication bias (eg, funnel plots) were not applicable or were not conducted.

#### Limitations of the Review Methodology

The transformation from quantitative evidence to qualitative descriptions may have resulted in inevitable information loss. The lack of evaluation of literature in languages other than Chinese and English may have led to the omission of some influencing factors. Although 2 researchers independently performed literature screening and data extraction to minimize subjective bias, the results of consistency testing were not reported. This shortcoming represents a methodological limitation regarding the transparency of the selection process.

### Conclusion

Guided by the CFIR, this study systematically analyzed the influencing factors of mHealth apps in kidney transplant care, which were categorized into 5 domains: intervention characteristics, outer setting, inner setting, characteristics of individuals, and process. The findings revealed that intervention characteristics constituted the most prominent influencing domain, while the outer setting and process demonstrated relatively fewer associated elements. Future research should further investigate the impact of these identified factors on mHealth apps in kidney transplant care. Additionally, health care providers should adopt multidimensional strategies targeting these determinants to optimize mHealth integration into clinical practice.

## Supplementary material

10.2196/84139 Multimedia Appendix 1 Search strategy in PubMed.

10.2196/84139 Checklist 1 PRISMA checklist.

## Abbreviations

CFIR: Consolidated Framework for Implementation Research
CNKI: China National Knowledge Infrastructure
mHealth: Mobile Health
MMAT: Mixed Methods Appraisal Tool
PRISMA: Preferred Reporting Items for Systematic Reviews and Meta-Analyses
PROSPERO: International Prospective Register of Systematic Reviews
