# Supplementary material for: Influencing Factors of Mobile Health Apps in Kidney Transplant Care: Systematic Review Using the Consolidated Framework for Implementation Research
Source: JMIR Med Inform. 2026 Mar 24;14:e84139. doi: 10.2196/84139 (PMC13012610; doi:10.2196/84139)
Supplement: Multimedia Appendix 1 [file medinform-v14-e84139-s001.docx]

| **No.** | **Search Details** |
| --- | --- |
| #1 | (kidney transplantation [MeSH Terms] OR kidney transplant* [Title/Abstract] OR renal transplant* [Title/Abstract] OR kidney graft* [Title/Abstract] OR renal graft* [Title/Abstract] OR kidney allograft* [Title/Abstract] OR renal allograft* [Title/Abstract] OR transplanted kidney [Title/Abstract] OR kidney homotransplantation* [Title/Abstract] OR renal homotransplantation* [Title/Abstract]) |
| #2 | (telemedicine [MeSH Terms] OR tele?medicine [Title/Abstract] OR mobile health [Title/Abstract] OR m?Health [Title/Abstract] OR mHealth [Title/Abstract] OR tele?health [Title/Abstract] OR telehealth [Title/Abstract] OR e?Health [Title/Abstract] OR eHealth [Title/Abstract] OR tele?rehabilitation [Title/Abstract] OR telerehabilitation [Title/Abstract] OR tele?monitoring [Title/Abstract] OR telemonitoring [Title/Abstract] OR video?conferencing [Title/Abstract] OR videoconferencing [Title/Abstract] OR online [Title/Abstract] OR technolog* [Title/Abstract] OR mobile information technolog* [Title/Abstract] OR electronic [Title/Abstract] OR digital [Title/Abstract] OR Internet based [Title/Abstract] OR app* [Title/Abstract] OR mobile application* [Title/Abstract] OR software [Title/Abstract] OR wearable electronic device* [Title/Abstract] OR WeChat [Title/Abstract] OR platform* [Title/Abstract] OR smartphone* [Title/Abstract]) |
| #3 | (motivator* [Title/Abstract] OR motivation* [Title/Abstract] OR enabler* [Title/Abstract] OR promote [Title/Abstract] OR drive [Title/Abstract] OR encourage [Title/Abstract] OR facilitator* [Title/Abstract] OR barrier* [Title/Abstract] OR obstacle* [Title/Abstract] OR challenge* [Title/Abstract] OR difficult* [Title/Abstract] OR issue* [Title/Abstract] OR influencing factor* [Title/Abstract] OR impact factor* [Title/Abstract] OR relative factor* [Title/Abstract] OR experience* [Title/Abstract] OR perception* [Title/Abstract] OR opinion* [Title/Abstract] OR feeling* [Title/Abstract] OR need* [Title/Abstract] OR attitude*) |
| #4 | #1 AND #2 AND #3 |

**Multimedia Appendix 1** Search strategy in PubMed
